# Supplementary material for: Breakdown of bulk-projected isotropy in surface electronic states of topological Kondo insulator SmB6(001)
Source: Nat Commun. 2022 Sep 23;13:5600. doi: 10.1038/s41467-022-33347-0 (PMC9508144; doi:10.1038/s41467-022-33347-0)
Supplement: Supplementary file 1 — Supplementary Information [file 41467_2022_33347_MOESM1_ESM.pdf]

Supplementary Information for: Breakdown of bulk-projected isotropy in surface electronic states of topological Kondo insulator $SmB_6(001)$

Yoshiyuki Ohtsubo,[1,2,3,*] Toru Nakaya,[3] Takuto Nakamura,[2,3] Patrick Le Fèvre,[4] François Bertran,[4] Fumitoshi Iga,[5] and Shin-Ichi Kimura[2,3,6,†]

[1]*National Institutes for Quantum Science and Technology, Sendai 980-8579, Japan*

[2]*Graduate School of Frontier Biosciences, Osaka University, Suita 565-0871, Japan*

[3]*Department of Physics, Graduate School of Science, Osaka University, Toyonaka 560-0043, Japan*

[4]*Synchrotron SOLEIL, Saint-Aubin-BP 48, F-91192 Gif sur Yvette, France*

[5]*Graduate School of Science and Engineering, Ibaraki University, Mito 310-8512, Japan*

[6]*Institute for Molecular Science, Okazaki 444-8585, Japan*

[*] y_oh@qst.go.jp

[†] kimura@fbs.osaka-u.ac.jp

SUPPLEMENTARY NOTE 1: HOMOGENEITY OF THE $\mathbf{SmB_6(001)}$-$p\mathbf{(2{\times}2)}$ SURFACE

It is known that the cleaved $SmB_6(001)$ surfaces exhibit numerous different surface domains with various topmost elements and inhomogeneous atomic structures, as observed by PES and STM [1–3]. However, we think that such patchy surface structure on cleaved surfaces is not dominant and is playing just a minor role on the $SmB_6(001)$-$p(2{\times}2)$ surface we prepared in this work. Let us discuss this issue here.

Firstly, it is already known that the inhomogeneous nature of the $SmB_6(001)$ surface is mostly removed by in-situ cleaning process by cycles of sputtering and annealing. For example, STM images and LEED pattern taken before and after the cleaning process exhibited a significant improvements of surface flatness and uniform surface structure (ref. [4]). While the miscut angle and annealing temperature of ours are different from those of ref. [4], it would be a natural expectation that the similar improvement to remove the patchy surface morphology also occurred in our samples.

Secondly, the sharp FCs around G-bar also supports the small (or nearly zero) area of inhomogeneity on our surface. Most of the ARPES studies performed on the cleaved surface exhibited just a blurred feature around there. This could be understood by supposing that the patchy surface structures behave as a random perturbation to TSS and thus the averaged ARPES data results in a broad hump as observed. The oval-shaped S1 is always observed even in such occasions, perhaps because it is not sensitive to surface perturbation comparing with the other TSSs around G-bar. This assumption is not so strange, if the wavefunction of S1 does not have large amplitude around the topmost surface but rather deeper layers. In contrast, in our case, all the three FCs were observed sharply. The peak widths of S2 and S3 around G-bar are nearly the same as that of S1, as shown in Fig. 3. It suggests that the broadening of S2 and S3 from surface patches discussed above has almost negligible contribution and the peak width of surface bands there are simply determined by k resolution of the ARPES equipment.

Thirdly, the $p(2{\times}2)$ surface superstructure could be reproduced three times, even after a few months under ambient condition. It means that the current surface preparation method removes surface contamination as well as disorders from ambient contamination repeatedly. It is unrealistic to assume that all the surface "patches" are robust against

such ambient pressure as well as the removal of surface layers by Ar ion sputtering in our preparation method. Therefore, we think the "patches" could exist on the surface only after the first polish of the substrate crystal, but should be removed during the repeated surface contamination (in the air) and cleaning processes.

Based on these points, we are sure that the $SmB_6(001)$-$p(2\times2)$ surface prepared in this work is free from the surface inhomogeneity.

SUPPLEMENTARY NOTE 2: PHOTOEXCITATION PROCESS OF ARPES

It is known that ARPES intensity often differs from the simple distribution of the density of initial (ground) states, because the observed electron belongs to the final state, free electron in vacuum and thus the photoexcitation process plays an important role upon it. Actually, evident intensity modulations at ARPEC FCs were reported in both bulk [5] and surface [6, 7] systems. This effect is called photoemission structure factor (PSF), as an analogy from those among diffraction experiments. Therefore, it is worth to be discussed whether the observed different intensities of S1 and S1' could be attributed to such artificial effects or not.

To consider this point, it should be noted that PSF appears between different, nonequivalent SBZs. For example, a metallic surface state on Ag/Si(111)-$(\sqrt{3}\times\sqrt{3})R30^\circ$ was observed at the 2nd SBZ but not at the 1st zone [7]. In addition, the photon-incident and photoelectron-detection planes also play a significant role, as the two-fold, not six, ARPES constant energy contours observed in graphite [5]. However, even after including such effect, ARPES intensities from the equivalent SBZ with the common photon-incident and photoelectron detection planes must be identical, as shown both from computational and experimental results [5, 7].

In the current case, $SmB_6(001)$-$p(2\times2)$, PSF rationalizes the absence of the FCs around $\bar{\mathrm{M}}$ folded by $p(2\times2)$ surface superstructure for example, because the $p(2\times2)$ SBZ around $\bar{\mathrm{M}}$ is the 3rd zone, different from the 1st (around $\bar{\Gamma}$) or 2nd (around $\bar{\mathrm{X}}$) zones. On the other hand, tiny intensity from S1' cannot be derived from it, because the SBZs around $\bar{\mathrm{X}}$ and $\bar{\mathrm{Y}}$ are equivalent 2nd zones, if one suppose four-fold rotation symmetry. Note that the experimental geometry for S1 and S1' is also equivalent with the common photon-incident and photoelectron-detection planes as depicted in Fig. 2c in the main text. For further

evidence, we measured the ARPES FC of $SmB_6(001)$-$p(2\times2)$ with different ARPES geometry as shown in supplementary Fig. 3. Here, the common photon-incident and photoelectron-detection planes are rotated 45° from those of Fig. 2. From supplementary Fig. 3, one can find that the S1 FC is still intense while those of S1' are very weak. Intensity modulations for $\pm k_y$ is because of circular dichroism (CD), since we measured this FC only by right-handed circularly polarized photons; CD does not justify the absence of S1', since it disappears at both signs of k_y. It is a smoking-gun evidence that PSF plays just a minor role on the disappearance of S1', strongly supporting that the difference between S1 and S1' are from the surface atomic structure.

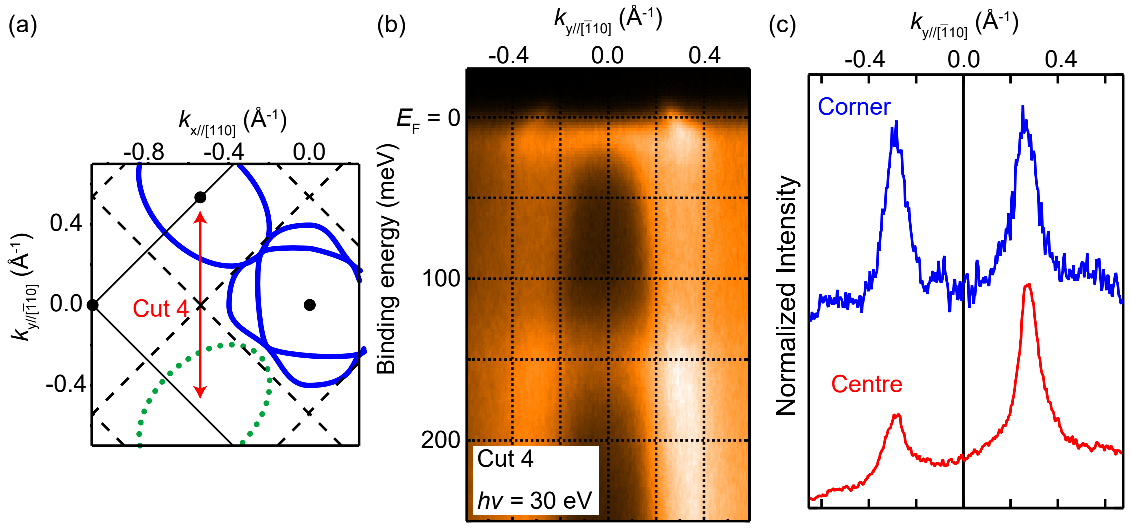

Supplementary Fig. 1. (a) Schematic drawing of the surface Fermi contours (FCs) observed by angle-resolved photoelectron spectroscopy (ARPES) (the same setup as that shown in Fig. 2c in the main text) together with the arrow indicating the position where the ARPES data in b and c were taken. (b) ARPES intensity plot taken with linearly polarized photons ($h\nu = 30$ eV) at 14 K. (c) Momentum distribution curves (MDCs) of the ARPES intensity plots at E_F along Cut 4 indicated in (a) at the sample centre (lower curve) and corner (upper curve). The peaks correspond to S1 and S1' defined in the main text. At the corner of the sample, the vicinal miscut angle is different from that in the rest of the area because of the edge rounding effect during the polishing process. Since the other measurement conditions are the same for the MDCs, this intensity difference between S1 and S1' should come from the area difference between surface domains, with the majority (minority) domains corresponding to S1 (S1').

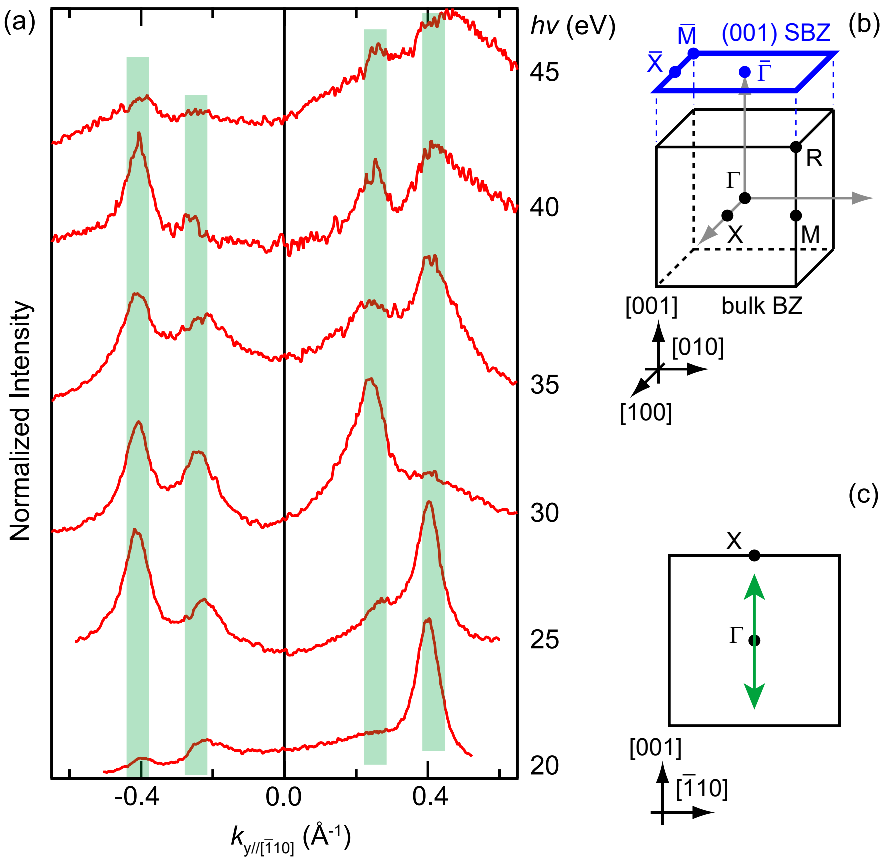

Supplementary Fig. 2. (a) ARPES MDCs along $\bar{\Gamma}$-$\bar{\mathrm{M}}$ (Cut 3 defined in the main text) taken with linearly polarized photons at 13 K. Fat lines indicate the measured peaks without significant dispersion along $k_{z//[001]}$, corresponding to surface FCs S2 and S3. The incident photon energies range from 20 to 45 eV. (b) Schematic drawing of the bulk and (001) surface Brillouin zones (BZ) of SmB_6. (c) Measured $k_{z//[001]}$ range of (a) in the bulk BZ assuming the inner potential of 10 eV. Photon energies of 18-45 eV (data shown in (a) and those with $h\nu = 18$ eV shown in the main text (Fig. 3d)) are used for calculating $k_{z//[001]}$ values. The initial and final positions of the measured range (the arrowheads) are placed as the worst case, where the inversion at the centre of the BZ doubles the measured range. The current energy range corresponds to the wavevector of 1.2 Å^{-1} along the surface normal ([001]) with this assumption. The reciprocal lattice vector along [001] of SmB_6 is 1.5 Å^{-1}, and thus, the measured range covers ∼80 % of the bulk BZ, even in this worst case. Together with the metallic character, in contrast to the insulating bulk electronic states at this temperature, the observed bands are quite likely to be the 2D surface states.

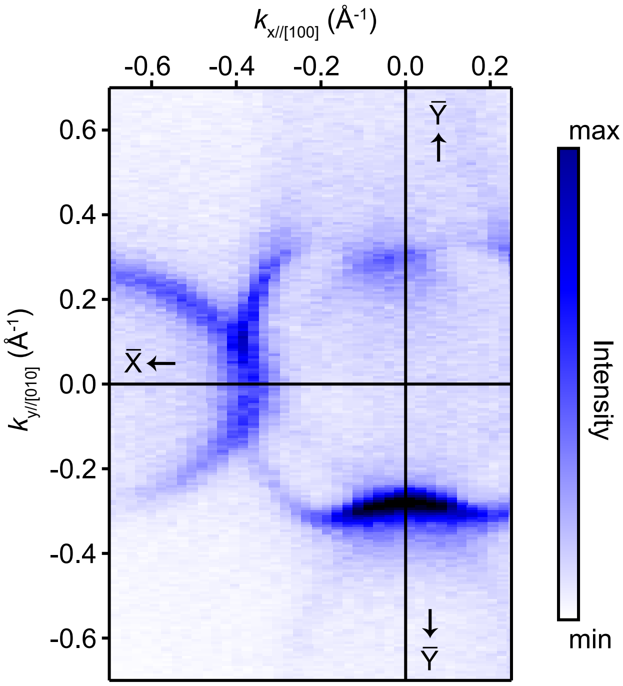

Supplementary Fig. 3. Fermi contours (FCs) obtained by ARPES taken with circularly polarized photons (right handed, $h\nu = 35$ eV) at 13 K. The photon incident plane is around (010).

Supplementary Tab. I. Fermi velocities (v_F) and areas of FCs (A) of each TSS. v_F for S1 (S2 and S3) is estimated from Cut 1 (Cut 3) in Fig. 3 taken with $h\nu = 35$ (18) eV. A are listed in two units so that they could be easily compared with the data from de Haas van Alphen measurements.

	v_F (eV Å)	A (Å^{-2})	A (kT)
S1	-0.25	0.30	3.1
S2	1.9	0.42	4.4
S3	-0.27	0.24	2.5

SUPPLEMENTARY REFERENCES

[1] Hlawenka, P. *et al.* Samarium hexaboride is a trivial surface conductor. *Nature Communications* **9**, 517 (2018).

[2] Kotta, E. *et al.* Metallic chemical potentials in an insulating topological state. *preprint at Research Square* (2022). URL https://doi.org/10.21203/rs.3.rs-1239795/v1.

[3] Rößler, S. *et al.* Hybridization gap and fano resonance in smb6. *Proceedings of the National Academy of Sciences* **111**, 4798–4802 (2014).

[4] Miyamachi, T. *et al.* Evidence for in-gap surface states on the single phase SmB_6(001) surface. *Scientific Reports* **7**, 12837 (2017).

[5] Nishimoto, H. *et al.* Two-dimensional angular distribution of photoelectrons of single-crystal graphite. *Journal of Physics: Condensed Matter* **8**, 2715–2732 (1996).

[6] Rotenberg, E. *et al.* Indium $\sqrt{7} \times \sqrt{3}$ on Si(111): A Nearly Free Electron Metal in Two Dimensions. *Phys. Rev. Lett.* **91**, 246404 (2003).

[7] Hirahara, T., Matsuda, I. & Hasegawa, S. Photoemission Structure Factor Effect for Fermi Rings of the Si(111)$\sqrt{3} \times \sqrt{3}$-Ag Surface. *e-Journal of Surface Science and Nanotechnology* **2**, 141–145 (2004).
